# Supplementary material for: Molecular mechanism of setron-mediated inhibition of full-length 5-HT3A receptor
Source: Nat Commun. 2019 Jul 19;10:3225. doi: 10.1038/s41467-019-11142-8 (PMC6642186; doi:10.1038/s41467-019-11142-8)
Supplement: Supplementary file 1 — Supplementary Information [file 41467_2019_11142_MOESM1_ESM.pdf]

Supplementary Information

Molecular mechanism of setron-mediated inhibition of full-length 5-HT_{3A} receptor

Sandip Basak, Yvonne Gicheru et al.

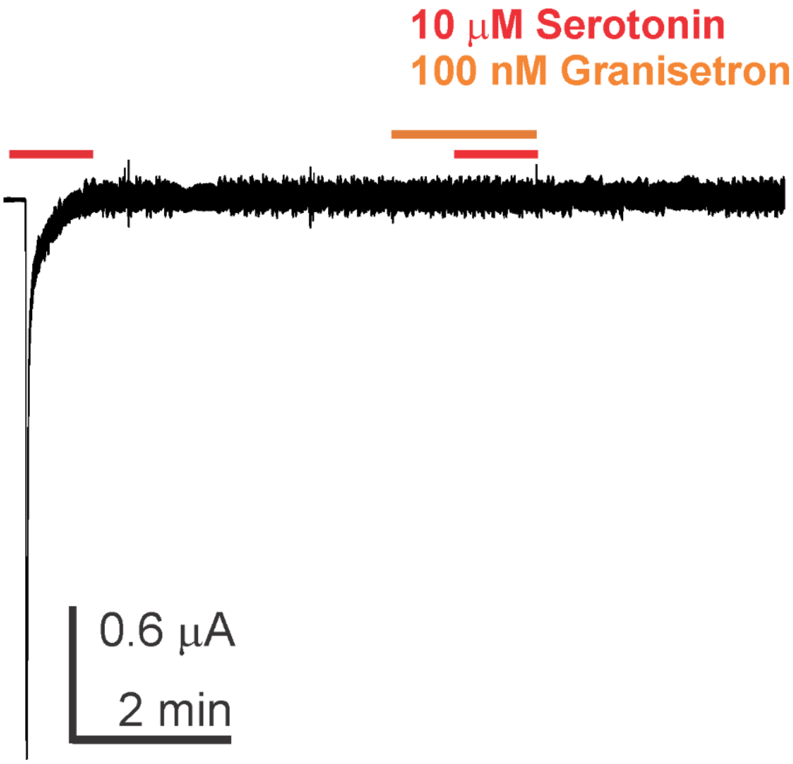

Supplementary Figure 1. Effect of 100 nM granisetron on 5-HT$_{3A}$R currents. TEVC recordings (at -60mV) for wild type 5-HT$_{3A}$R in the presence of 10 μM serotonin, upon pre-application of 100 nM granisetron, and co-application of 10 μM serotonin and 100 nM granisetron.

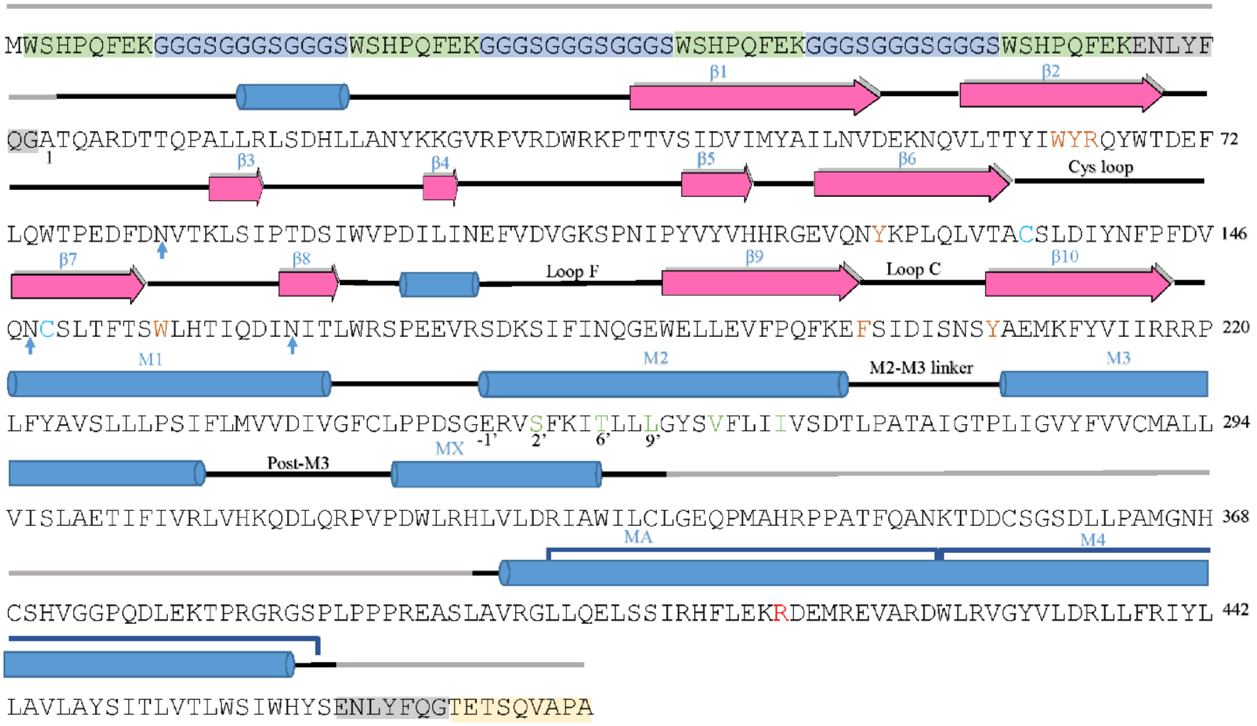

Supplementary Figure 2. Sequence of mouse 5-HT$_{3A}$R used in the cryo-EM study. Full length mouse 5-HT$_{3A}$R sequence used in the cryo-EM study. The sequence also includes strep-tags (green), linker regions (blue), TEV cleavage site (gray), and 1D4-tag (yellow). Secondary structural elements are indicated above the sequence. Regions in the M3-M4 linker indicated in gray color are not seen in the final refined structure. Glycosylation sites are marked as blue arrows. Key residues involved in serotonin binding sites are highlighted in brown color. Cysteines present in the cys-loop are shown as cyan color. Pore-facing residues in M2 are shown in green color. Arg416 in the ICD is shown in red.

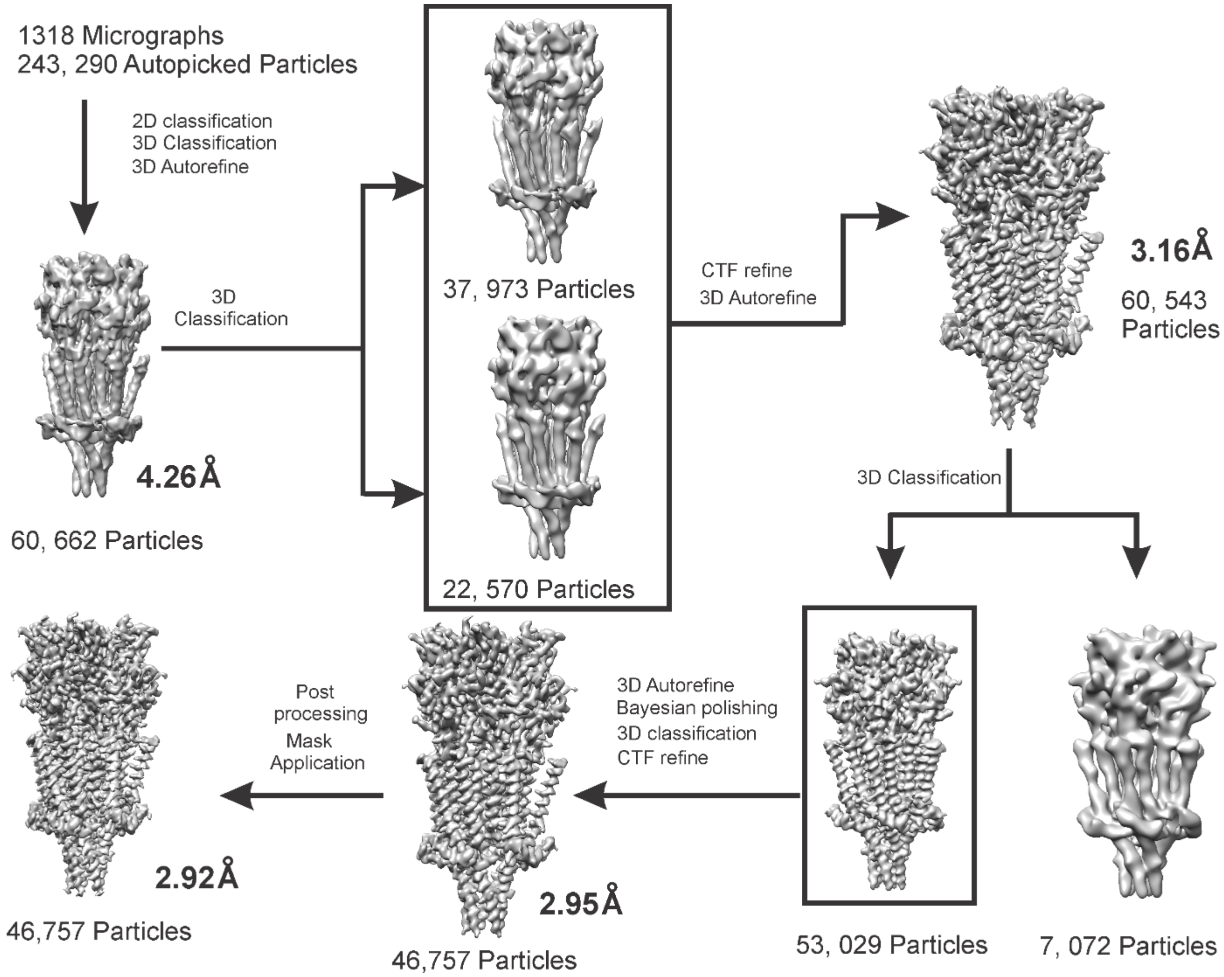

Supplementary Figure 3. Data Processing workflow. A schematic representation of the steps followed in data processing leading to 2.92 Å reconstruction. Classes within the box used for further processing.

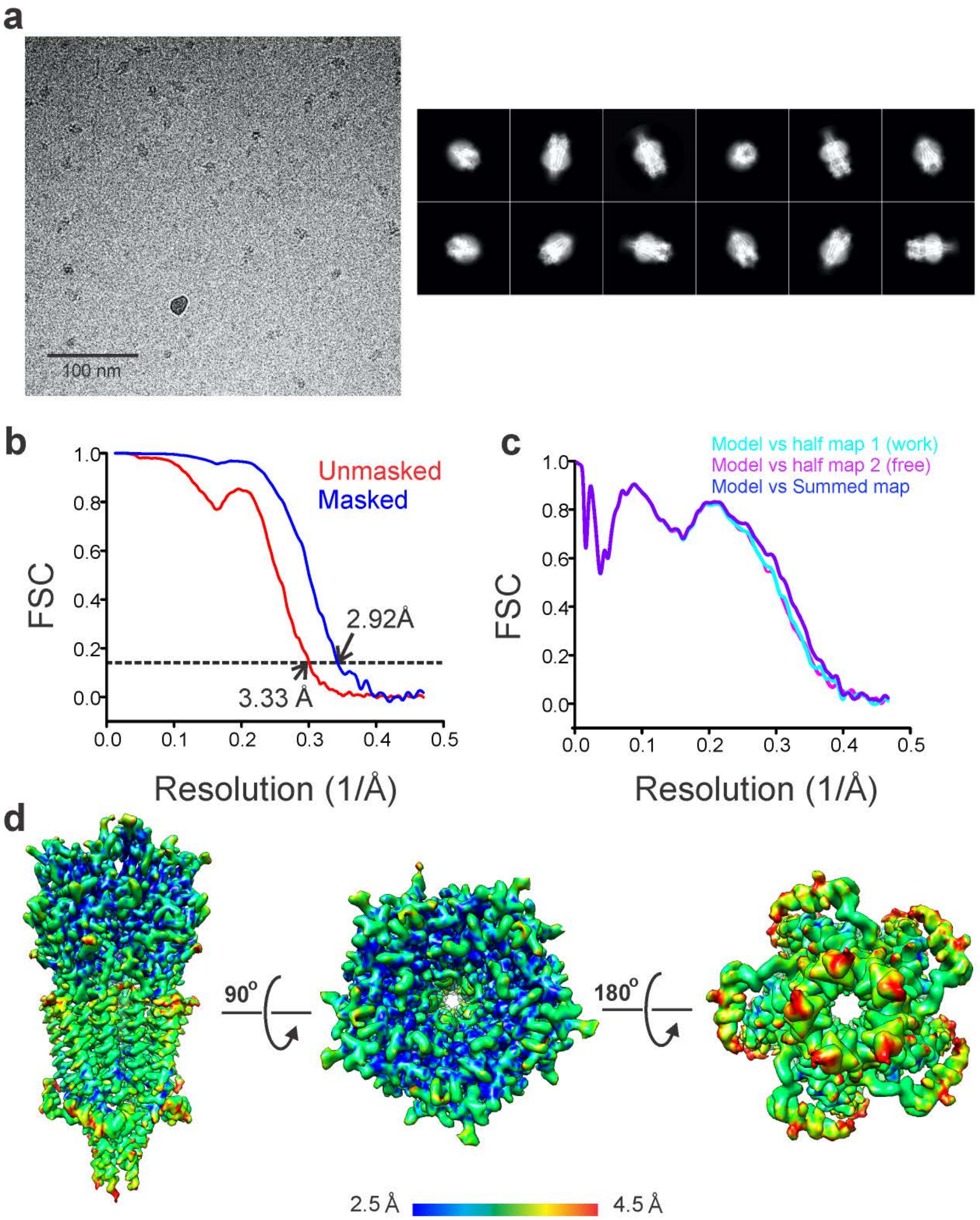

Supplementary Figure 4. Estimation of resolution and validation of the models. **a**. A representative micrograph of 5-HT$_{3A}$R incubated with 100 μM granisetron in vitreous ice (left). 2D classes showing various orientations used for 3D reconstruction (right). **b**. Fourier shell correlation curves before (red) and after (blue) the mask application in RELION. The dashed line indicates FSC of 0.143. **c**. For validation, FSC curves of the refined model versus summed map, refined model versus half map 1 (used for refinement), and refined model versus half map 2 (not used for refinement) were calculated. **d**. Different views of local resolution of 5-HT$_{3A}$R-granisetron reconstruction was estimated using the ResMap program.

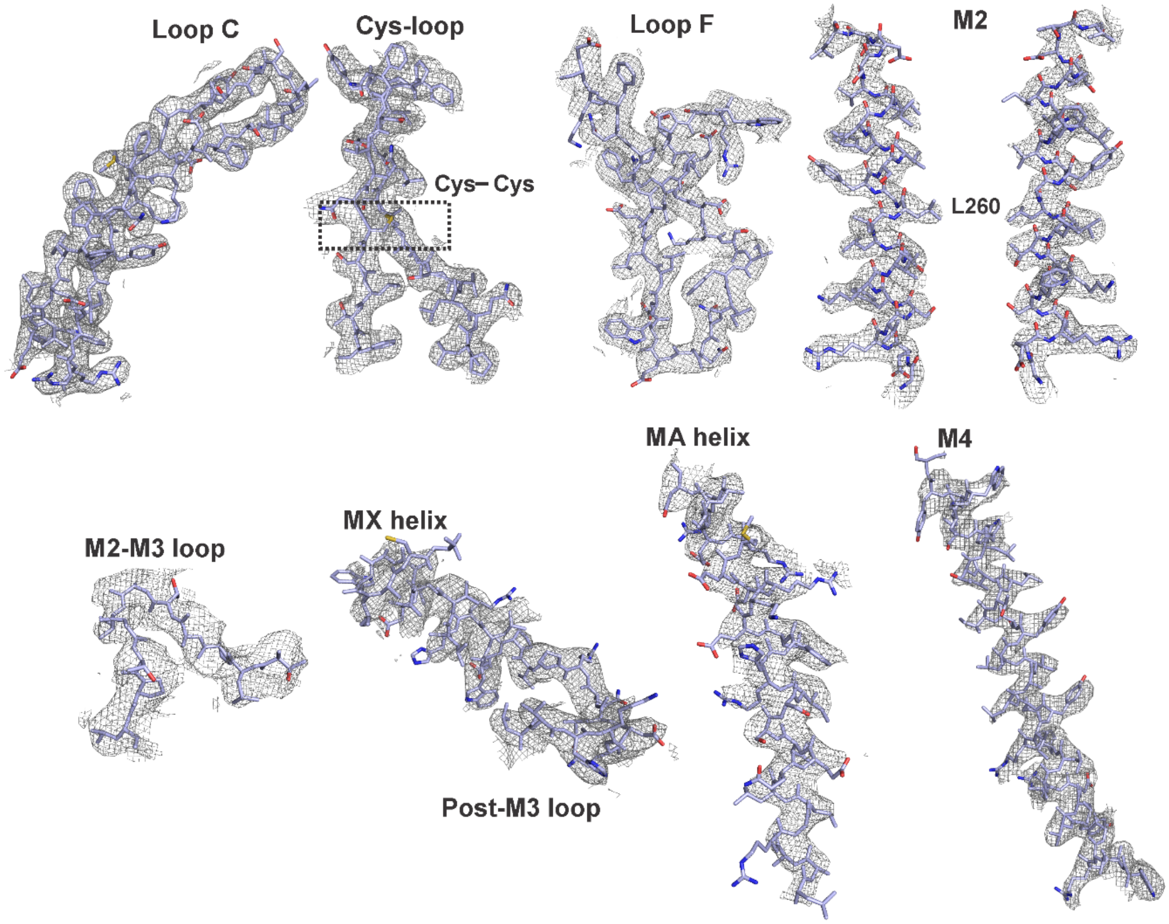

Supplementary Figure 5. Map correlation of granisetron-5-HT3AR structure. Validation of the various regions of the model (shown in stick representation) and corresponding density map (mesh) around the residues are shown here. Residues are represented as sticks. The depicted regions in 5-HT3AR-granisetron and the corresponding contour levels: Cys loop (8.2 σ), loop C (8.2 σ), loop F (8.0 σ), M2 (8.2 σ), M2-M3 (6.5 σ), M4 (8.2 σ), MX helix (8.0 σ), and MA helix (8.2 σ). The boxed region highlights the cysteine disulfide-bridge in cys-loop.

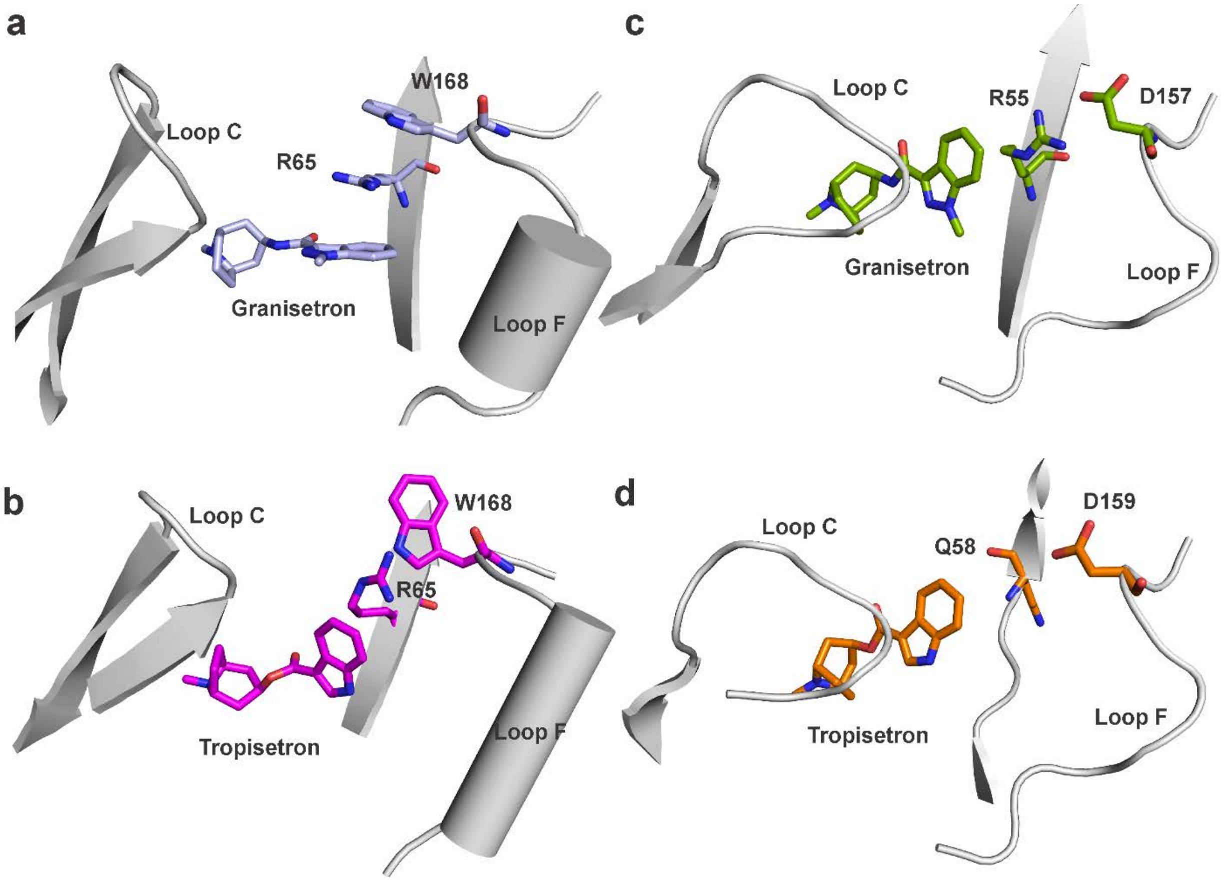

Supplementary Figure 6. Structural differences between granisetron and tropisetron binding poses in 5-HT$_{3A}$R and AChBP structures. a. In the 5-HT$_{3A}$R-granisetron structure, the indazole ring in granisetron lies flat in the binding site allowing for a potential cation-pi stacking interaction between Arg65, granisetron and Trp168 in loop F. **b.** In the 5-HT$_{3A}$R-tropisetron structure[1], the indole ring is tilted upwards (by 86.2° with respect to granisetron, measured at C_{12}-C_{14} bond). Trp168 sidechain is tilted away from the binding site and Arg65 may adopt a different rotameric orientation (although the density for this region is not clear). **c.** The granisetron-binding pose in the AChBP-5-HT$_{3A}$R chimeric mutant[2]. Positions equivalent to R65 and W168 in 5-HT$_{3A}$R are shown. **d.** The tropisetron-binding pose as seen in the structure of AChBP crystal structure[3]. Positions equivalent to R65 and W168 in 5-HT$_{3A}$R are shown.

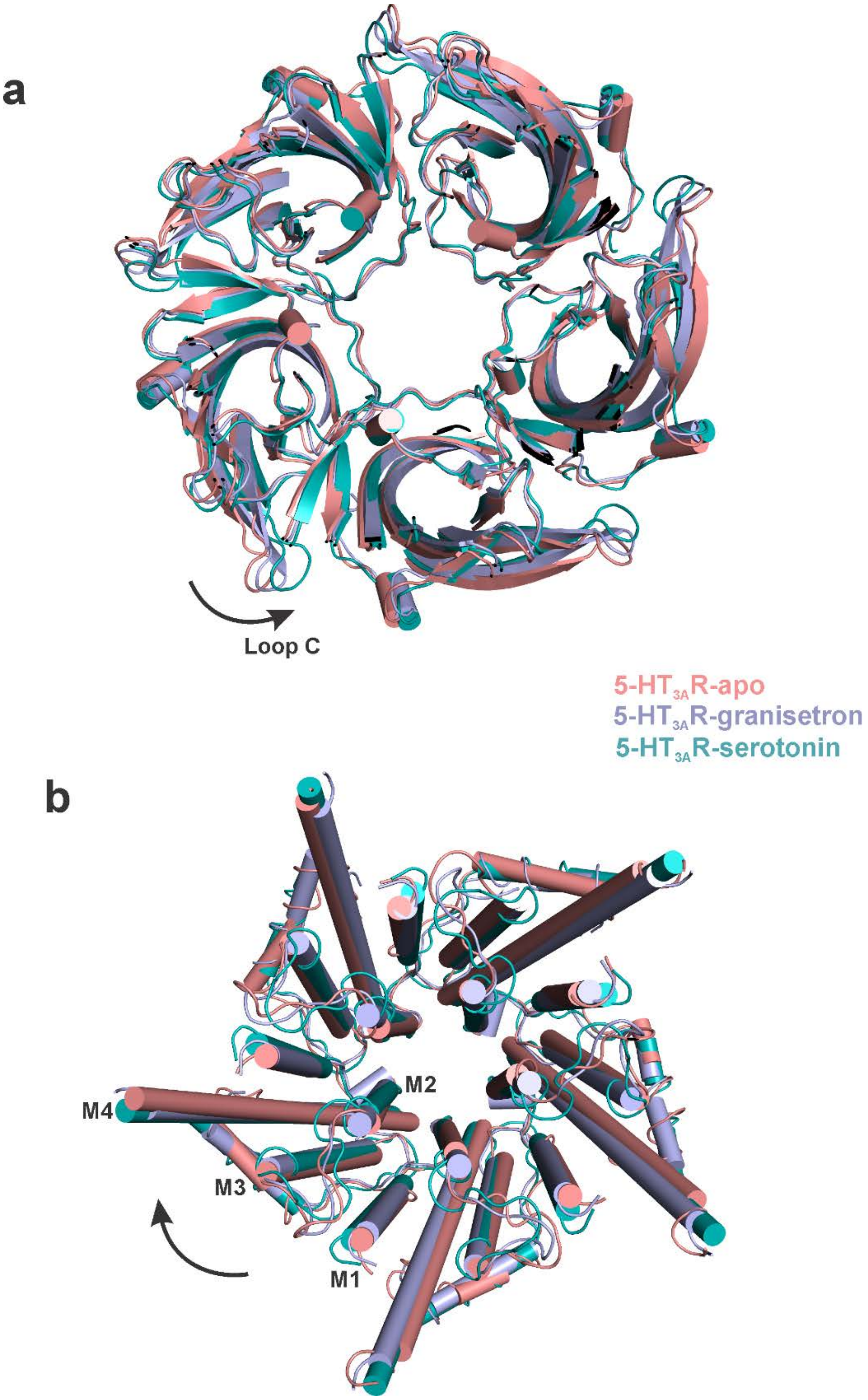

Supplementary Figure 7. Alignment of apo and ligand-bound structures of 5-HT$_{3A}$R. An alignment of 5-HT$_{3A}$R-apo, 5-HT$_{3A}$R-granisetron, 5-HT$_{3A}$R-serotonin structures. **a.** A view of the ECDs from the extracellular end when aligned with respect to the TMDs. **b.** A view of the TMDs from the extracellular end when aligned with respect to the ECDs. The arrows show the putative direction of displacements among the three structures.

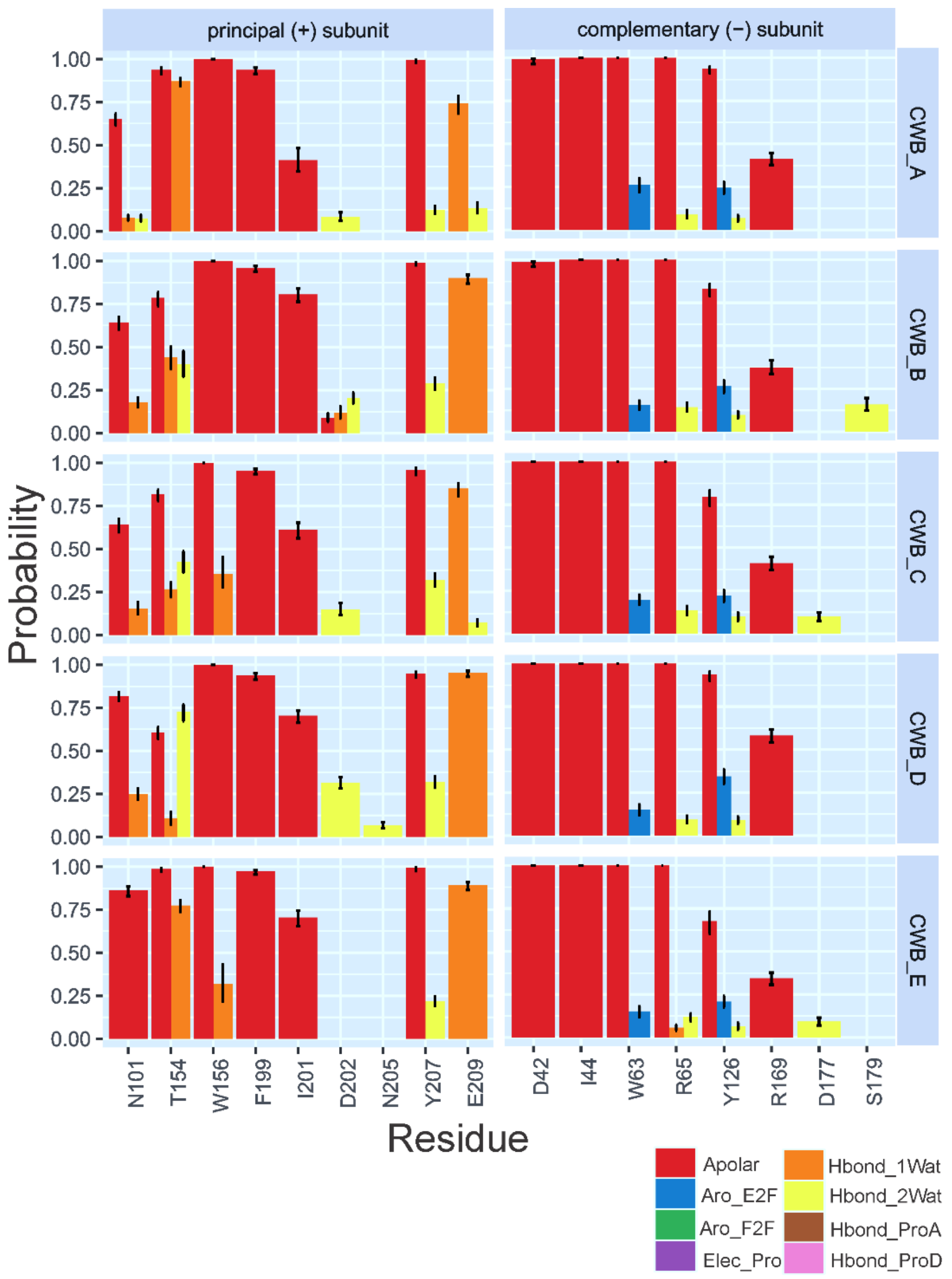

Supplementary Figure 8. Molecular interactions between granisetron and ligand-binding residues during simulation. 5-HT_{3A}R-granisetron interaction fingerprints calculated for each ligand-protein complex (labelled CWB_A-CWB_E) from 100 ns production simulations. Nine interaction types are calculated: apolar (hydrophobic), face-to-face aromatic (Aro_F2F), edge-to-face aromatic (Aro_E2F), hydrogen bond with the protein as hydrogen bond donor (Hbond_ProD), hydrogen bond with the protein as hydrogen bond acceptor (Hbond_ProA), electrostatic with the protein positively charged (Elec_ProP), electrostatic with the protein negatively charged (Elec_ProN,), one-water mediated and two-water mediated hydrogen bond interactions. Ligand-protein interactions are limited to those formed with 5-HT_{3A}R side chains. Only interactions with average probability above 5% are displayed.

```
Forward: 5′-ACAATGAGCTCATGCGGCTCTGCATCCCGC-3′
Reverse: 5′-TGGCTGGATCCTTAAGAATAATGCCAAATGGACCAG-3′
```

Supplementary Table 1. Primers for cloning of 5-HT_{3A}R into pTLN vector for oocyte expression using SacI and BamHI restriction sites.

```
>5HT3AR-Mouse_codon_optimized
ACTAGTATGCGCCTGTGCATCCCTCAGGTGCTGCTGGCTCTGTTCCTGTCCATGCTGACTGCTCCTGGAGAGGG
CAGCCGCCGTAGGTGGAGCCACCCGCAGTTCGAAAAAGGTGGTGGTTCAGGTGGTGGTTCAGGTGGTGGTTCAT
GGAGCCACCCGCAGTTCGAAAAAGGTGGTGGTTCAGGTGGTGGTTCAGGTGGTGGTTCATGGAGCCACCCGCAG
TTCGAAAAAGGTGGTGGTTCAGGTGGTGGTTCAGGTGGTGGTTCATGGAGCCACCCGCAGTTCGAAAAAGAGAA
CCTGTACTTCCAGGGCGCTACTCAGGCCCGCGACACCACTCAGCCTGCTCTGCTGCGTCTGTCTGACCACCTGC
TGGCCAACTACAAGAAGGGCGTGCGTCCCGTCAGGGACTGGAGAAAGCCAACCACTGTGTCAATCGACGTCATC
ATGTACGCTATCCTGAACGTGGACGAGAAGAACCAGGTCCTGACCACTTACATCTGGTACAGGCAGTACTGGAC
TGACGAGTTCCTGCAGTGGACCCCCGAAGACTTCGACAACGTGACTAAGCTGTCCATCCCTACCGACAGCATCT
GGGTCCCCGACATCCTGATCAACGAGTTCGTGGACGTCGGCAAGTCCCCTAACATCCCCTACGTGTACGTCCAC
CACCGTGGAGAAGTGCAGAACTACAAGCCACTGCAGCTGGTCACCGCCTGCAGCCTGGACATCTACAACTTCCC
TTTCGACGTGCAGAACTGCTCTCTGACCTTCACTTCATGGCTGCACACTATCCAGGACATCAACATCACCCTGT
GGCGCTCCCCTGAGGAAGTGAGATCTGACAAGTCAATCTTCATCAACCAGGGAGAGTGGGAACTGCTGGAGGTC
TTCCCACAGTTCAAGGAATTCTCAATCGACATCTCTAACTCATACGCTGAGATGAAGTTCTACGTGATCATCCG
CCGTAGGCCCCTGTTCTACGCCGTCTCCCTGCTGCTGCCAAGCATCTTCCTGATGGTGGTCGACATCGTGGGTT
TCTGCCTGCCTCCCGACTCTGGCGAACGTGTCTCATTCAAGATCACTCTGCTGCTGGGATACTCTGTGTTCCTG
ATCATCGTCTCAGACACCCTGCCAGCTACTGCCATCGGCACCCCTCTGATCGGAGTGTACTTCGTGGTCTGCAT
GGCTCTGCTGGTCATCTCCCTGGCCGAAACCATCTTCATCGTGAGGCTGGTCCACAAGCAGGACCTCCAGCGCC
CTGTGCCTGACTGGCTGCGCCACCTGGTCCTGGACCGTATCGCTTGGATCCTGTGCCTGGGTGAACAGCCTATG
GCCCACAGGCCACCTGCTACTTTCCAGGCCAACAAGACCGACGACTGCTCCGGAAGCGACCTGCTGCCTGCTAT
GGGTAACCACTGCTCTCACGTGGGTGGCCCACAGGACCTGGAGAAGACCCCAAGAGGTAGGGGTTCCCCTCTGC
CCCCACCTAGGGAAGCTAGCCTGGCCGTGAGAGGTCTGCTGCAGGAGCTGTCCAGCATCCGCCACTTCCTGGAA
AAGCGCGACGAGATGCGTGAAGTCGCCAGGGACTGGCTGAGAGTGGGCTACGTCCTGGACCGCCTGCTGTTCCG
TATCTACCTGCTGGCTGTGCTGGCCTACTCCATCACCCTGGTCACTCTGTGGTCCATCTGGCACTACAGCGAAA
ACCTGTATTTTCAGGGCACAGAGACCAGCCAAGTCGCGCCTGCGTAAGCGTTACATCACCATCACCATCACCAT
CACTAAACAGAGACCAGCCAAGTCGCGCCTGCGGGTGGTGGTTCAGGTGGTGGTTCAGGTGGTGGTTCAACAGA
GACCAGCCAAGTCGCGCCTGCGTAAGGTACC
```

Supplementary Table 2. Codon optimized mouse 5-HT_{3A}R sequence

Supplementary References

1 Polovinkin, L. *et al.* Conformational transitions of the serotonin 5-HT3 receptor. *Nature* **563**, 275-279, (2018).

2 Kesters, D. *et al.* Structural basis of ligand recognition in 5-HT3 receptors. *EMBO Rep* **14**, 49-56, (2013).

3 Hibbs, R. E. *et al.* Structural determinants for interaction of partial agonists with acetylcholine binding protein and neuronal alpha7 nicotinic acetylcholine receptor. *Embo J* **28**, 3040-3051 (2009).
