# Supplementary material for: Molecular mechanism of setron-mediated inhibition of full-length 5-HT3A receptor
Source: Nat Commun. 2019 Jul 19;10:3225. doi: 10.1038/s41467-019-11142-8 (PMC6642186; doi:10.1038/s41467-019-11142-8)
Supplement: Supplementary file 3 — Reporting Summary [file 41467_2019_11142_MOESM3_ESM.pdf]

nature research

Corresponding author(s): Sudha Chakrapani

Last updated by author(s): Jun 17, 2019

Reporting Summary

Nature Research wishes to improve the reproducibility of the work that we publish. This form provides structure for consistency and transparency in reporting. For further information on Nature Research policies, see Authors & Referees and the Editorial Policy Checklist.

Statistics

For all statistical analyses, confirm that the following items are present in the figure legend, table legend, main text, or Methods section.

n/a	Confirmed	
☐	☒	The exact sample size (*n*) for each experimental group/condition, given as a discrete number and unit of measurement
☐	☒	A statement on whether measurements were taken from distinct samples or whether the same sample was measured repeatedly
☐	☒	The statistical test(s) used AND whether they are one- or two-sided<br>*Only common tests should be described solely by name; describe more complex techniques in the Methods section.*
☒	☐	A description of all covariates tested
☒	☐	A description of any assumptions or corrections, such as tests of normality and adjustment for multiple comparisons
☐	☒	A full description of the statistical parameters including central tendency (e.g. means) or other basic estimates (e.g. regression coefficient) AND variation (e.g. standard deviation) or associated estimates of uncertainty (e.g. confidence intervals)
☐	☒	For null hypothesis testing, the test statistic (e.g. *F*, *t*, *r*) with confidence intervals, effect sizes, degrees of freedom and *P* value noted<br>*Give P values as exact values whenever suitable.*
☐	☒	For Bayesian analysis, information on the choice of priors and Markov chain Monte Carlo settings
☒	☐	For hierarchical and complex designs, identification of the appropriate level for tests and full reporting of outcomes
☒	☐	Estimates of effect sizes (e.g. Cohen's *d*, Pearson's *r*), indicating how they were calculated

Our web collection on statistics for biologists contains articles on many of the points above.

Software and code

Policy information about availability of computer code

Data collection	Clampfit 10.2, EPU from ThermoFisher
Data analysis	1. Drift correction: MotionCor version 2.1.1.0<br>2. CTF estimation: GCTF version 1.06<br>3. 2D and 3D Reconstruction, 3D refinement, post-processing: Relion Version 3.0<br>4. Local resolution estimation: Resmap version 1.1.4<br>5. Pore profile calculation: HOLE version 3.0<br>6. Model visualization: PyMOL version 2.0.4<br>7. 3D volume visualization: Chimera version 1.11.2<br>8. MRC to MTZ conversion: CCP4I version 7.0.057<br>9. Manual model building: Coot 0.8.9.1<br>10. Structure refinement: Phenix version 1.13-2998<br>11. MD simulations: Gromacs 2018.2, Gromacs 2016.3 patched with PLUMED 2.3.1 (for metadynamics)<br>12. Various CRYO-EM data conversion: EMAN version 2.2<br>13. Figure generation: CorelDraw version 20.1.0.708<br>14. Electrophysiology data analysis: Clampfit version 10.2<br>15. Electrophysiology plot generation: Origin Version b9.5.0.193

For manuscripts utilizing custom algorithms or software that are central to the research but not yet described in published literature, software must be made available to editors/reviewers. We strongly encourage code deposition in a community repository (e.g. GitHub). See the Nature Research guidelines for submitting code & software for further information.

Data

Policy information about availability of data

All manuscripts must include a data availability statement. This statement should provide the following information, where applicable:

- Accession codes, unique identifiers, or web links for publicly available datasets
- A list of figures that have associated raw data
- A description of any restrictions on data availability

The coordinates of the 5-HT3AR-granisetron structure and the Cryo-EM map have been deposited under PDB ID: 6NP0; EMBD ID: EMD-0469 with the wwPDB and EMDB

Field-specific reporting

Please select the one below that is the best fit for your research. If you are not sure, read the appropriate sections before making your selection.

☒ Life sciences ☐ Behavioural & social sciences ☐ Ecological, evolutionary & environmental sciences

For a reference copy of the document with all sections, see nature.com/documents/nr-reporting-summary-flat.pdf

Life sciences study design

All studies must disclose on these points even when the disclosure is negative.

Sample size	For electrophysiological recordings, the wt and mutants were recorded from independently injected oocytes. The n values refers to each oocyte and is mentioned in the figure legend.
Data exclusions	N/A
Replication	10 independent metadynamics simulations were completed.
Randomization	N/A
Blinding	N/A

Reporting for specific materials, systems and methods

We require information from authors about some types of materials, experimental systems and methods used in many studies. Here, indicate whether each material, system or method listed is relevant to your study. If you are not sure if a list item applies to your research, read the appropriate section before selecting a response.

Materials & experimental systems

n/a	Involved in the study	
☒	☐	Antibodies
☒	☐	Eukaryotic cell lines
☒	☐	Palaeontology
☒	☐	Animals and other organisms
☒	☐	Human research participants
☒	☐	Clinical data

Methods

n/a	Involved in the study	
☒	☐	ChIP-seq
☒	☐	Flow cytometry
☒	☐	MRI-based neuroimaging
